# Insight into the changes of European agriculture during the age of Baroque and enlightenment: Interdisciplinary survey of manor farmyard Švamberk (Czech Republic)

**DOI:** 10.1016/j.heliyon.2024.e40916

**Published:** 2024-12-04

**Authors:** Libor Vobejda, Tereza Šálková, Yulia V. Erban Kochergina, Jan Altman, Zuzana Thomová

**Affiliations:** a Institute of Archaeology, Faculty of Arts, University of South Bohemia in České Budějovice, Branišovská 31a, CZ, 370 05, České Budějovice, Czech Republic; b Division of Geochemistry and Laboratories, Czech Geological Survey, Geologická 6, CZ, 152 00, Prague, Czech Republic; c Institute of Botany of the Czech Academy of Sciences, Dukelská 135, 379 01, Třeboň, Czech Republic; d Faculty of Forestry and Wood Sciences, Czech University of Life Sciences, Prague, Kamýcká 129, 165 21, Prague 6, Suchdol, Czech Republic; e Department of Archaeology, South Bohemian Museum in České Budějovice, Fráni Šrámka 4, CZ, 370 05, České Budějovice, Czech Republic

**Keywords:** Post medieval archaeology, Landscape, Agriculture, Neophytes, Dendroprovenance, Vault infills, Archaeootany, Dendrochronology

## Abstract

Following European exploration of the Americas in the late 15th century, new plants rapidly spread across Europe. Simultaneously, plants from Asia and Africa arrived. Initially, they were grown in ornamental gardens but later became integral to major production centres, significantly transforming European agriculture. Neophytes gained prominence during a period of rapid economic progress in central Europe, and many have been cultivated since the 17th century. Their importance is documented through written sources and archaeobotanical findings.

This study of the manor farm Švamberk (Czechia) highlights how multidisciplinary research of agricultural production centres is crucial for understanding pre-industrial landscapes and the environmental impact of early modern societies. Agriculture's development correlates with changes in a landscape now suppressed by industrial interventions, yet key to sustainable development.

Plant remains in vault infills and roofs at Švamberk farmstead were dated using dendrochronology, with 99 samples and 81,892 plant macroremains analysed. Dendrochronological and strontium isotope analyses trace forestry and timber trade over time.

Timber felled in the 17th century was likely local, but by the late 18th century, timber came via complex transportation from southern Bohemia. Primary crops were grains, oilseeds, and vegetables, with evidence of exotic species like maize, tobacco, sunflowers (native to the Americas), sorghum (native to Africa), Parthenocissus, and Chinese thuja (native to Asia), some of the oldest archaeological evidence of their cultivation in central Europe.

## Highlights

• Archaeobotanical analysis of plant remains, combined with timber dating, reveals landscape changes during the modern era.
• Dendrochronological and dendroisotopic data indicate changes in forest management and the wood market of modern periods.
• New crops from the Americas were gradually introduced to European agriculture but remained limited until the 18th century.
• The heritage of the Baroque landscape, remains important despite being replaced by an industrialized landscape.

## Graphical abstract

**Figure:**
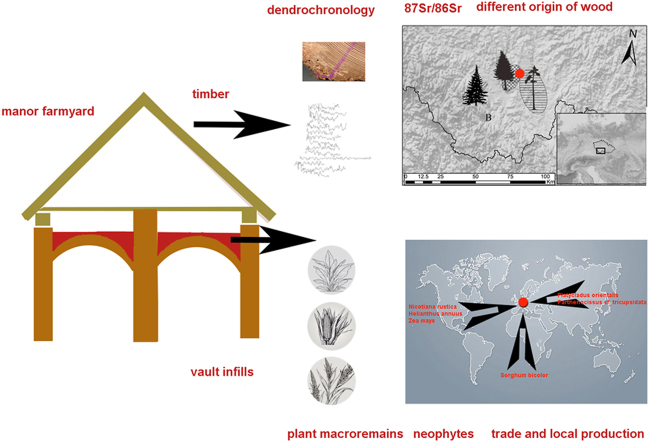

## 1 Introduction

A Significant portion of Earth's ecosystems has been under strong influence of anthropogenic factors since prehistory [[1], [2], [3]]. Many habitats were intentionally and unintentionally created or altered by human activity and by the introduction of new species [4].

Although definition of “landscape” varies widely across various disciplines, these habitats, along with various anthropogenic features (such as historical farm buildings), are important components of the rural landscape [[5], [6], [7]]. Industrialisation, which took place in the late 20th and early 21st centuries, significantly transformed European rural landscapes, among other things [8]. However, relics of the past rural landscapes are still common [7], and their protection and understanding are crucial for the sustainable development of future landscapes [9].

The origins of these structured landscapes can be traced back to the medieval period, but their modern form was established in the 17th and 18th centuries [10]. In some European countries, significant changes to field systems occurred due to collectivisation and the merging of fields in the 20th century [9,11,12]. Despite these changes, the forest management principles established in the 18th century have been maintained by foresters until the 21st century [13].

The character of the landscape in modern times was influenced by Baroque and later closely connected to the spread of Enlightenment ideas, which transformed perceptions of nature and its aesthetics [[13], [14], [15], [16], [17]]. Agriculture and forestry during this period were shaped by dominant capitalism, with the growing presence of newly discovered spices and crops (playing a crucial role) [[18], [19], [20]].

The period of economic growth following the Thirty Year's War (1618–1648) was characterised by intense competition between European countries. Many proxy and direct conflicts were waged over control of maritime trade [21], including the import of popular exotic goods. Central Europe could not benefit directly from Atlantic trade and was dependent on trading centres in the Mediterranean or the Baltic. The Netherlands, Habsburg Spain, and France, whose role was strengthened after their victory in the War of the Spanish Succession (1701–1714), were crucial for imports into central Europe [22]. In contrast, many Eastern European countries based their economies on the export of various cereals and wool to Western Europe [[23], [24], [25]].

The Age of Enlightenment^1^ is partly linked to the economic theory of mercantilism, which strongly discouraged imports in favour of exports.^2^ One of its aspects was the production of luxury goods and spices [26,27]. Eastern and central Europe were influenced to some extent by mercantilist doctrine, which found its way into various treatises and instructions [24]. Even though mercantilism favoured export and local use, the import of various exotic goods is well documented [19,28,29]. Initially, the nobility encountered exotic species primarily during their cavalier travels and through newspapers [30], and over time, they supported the breeding and cultivation of these species for various purposes [20,31]. The cultivation of some exotic crops in early modern Bohemia (now the Czech Republic) was no exception [19].

After the Thirty Years' War ended, central Europe was economically drained. This situation [32,33] along with the political changes following the conflict, caused a massive shift in the ownership of noble estates in Habsburg-held territories, particularly in the Kingdom of Bohemia [34]. Loyal catholic nobility acquired vast amounts of landed property, and these new landowners introduced various agricultural and economic innovations [15,33,[35], [36], [37]].

Similarly, the agricultural and market systems changed significantly compared to previous centuries [32,[37], [38], [39]]. In contrast to the high mediaeval period, new challenges emerged. Due to connections to the global trade network, international events began to influence local markets to a greater extent. As a result, the prices of agricultural products became less dependent on endogenous factors that could be predicted, such as local highs and lows in agricultural production [40,41].

Urbanisation and demographic decline led manor farms taking on a dominant role in agricultural production. These economic complexes provided commodities and products for the noble court, the local population of the dominion, and wider markets [38,41,42]. Corvée labour (mandatory labour of serfs) was reestablished to a greater extent in Bohemia and Moravia (now the Czech Republic) compared to western Europe [23,37,39,40].

The central European manorial system was a specific structure that originated in the medieval feudal economy. It involved the economic division of territorial units under the administration of a lord and court officials [23,41]. It was common for multiple manors to be owned by a single lord. The Area of the manor was a sustainable network of manor farms with varied segments of agricultural production [10,14,39,43]. This system effectively protected the population and the economic interests of the nobility against price fluctuation and economic crisis [23,33,41].

Each manor farm (also called: *Meierhof, Hof, Dvůr, Dvor* [23]) was a complex agricultural system that contained large areas of arable land, meadows for pasture and animal husbandry, forests and game preserves [38]. Some estates focused on fish production [44] supplementing the agricultural manor farms.

Forestry and logging in forested areas were of strategic importance [45]. Wood was used for charcoal burning, heating, and primarily for construction. Trees used for timber production were usually harvested near the construction sites [46,47]. In the case of a noble manor, the consumption of wood was enormous. Archival sources document the transport of short firewood and timber from the Šumava Mountains via the Vltava River. The logs were tied together at binding stations and floated further down the river. The Šumava logs were primarily unloaded from the river in České Budějovice, but partly also at other stations north of České Budějovice and in Prague, which was a destination for many rafts [48,49].

A wider pattern of forestry changes is observed across larger territories, but the causes and consequences related to the economy and environment remain unclear [50]. Although manor farms were similar in their main activities; namely the production of grains and cattle, their specialisations varied based on demographics, available resources, and management practices [33,41,42,51,52]. Thus, research concerning the broader agricultural activities that took place on the manor farms is needed to address following research questions: How could the farms interacted with the environment at various levels? What role did the manor farms play in the transformation of the landscape and agriculture? How did forestry and agriculture change during the modern era, and how can environmental archaeology methods be applied to address this question?

To study the origins of the structured rural landscape and the development of modern agriculture, we have number of tools at our disposal. Information about land use can, to some extent, be derived from forestry maps and maps of manors [53]. We also know the amount of crop production, the number of animals raised on the manor farms, and the accounts for building material. However, these written sources do not provide details about the character of the landscape in connection with the habitats present. Many produced and collected plants, mainly spices, medicinal plants, and minor crops, were unrecorded [10,15,30,52,54,55].

Archaeobotanical research from the early and high modern periods focuses on plant remains assemblages that are associated with cesspits and waste deposits from towns and palaces. Although these archaeological features contain a significant number of macroscopic remains (macroremains) of imported plants and provide insights into consumption patterns, they are not a suitable source of information for studying agricultural production [18,19]. The written accounts from noble courts and bookkeeping records [24,56] provide only partial information about what was produced.

Understanding the details of an interconnected network of farms can only be achieved through archaeological surveys of production centres (farmyards). However, direct evidence of the economic activities recovered during the archaeological survey needs to be evaluated using a multidisciplinary approach. Thus, an archaeobotanical survey allows us to reconstruct the ecological and economic aspects of past societies, as well as the history of vegetation and the character of the landscape [57].

We aim to disentangle the economic activities of one of the manor farms, namely Švamberk (South Bohemia), using a unique combination of dendrochronological and archaeobotanical approaches. We selected the relatively well-preserved manor farm of Švamberk, which was uniquely surveyed during its destruction. We propose to employ dendrochronological dating of the individual building timbers in conjunction with the analysis of a large quantity of plant macroremains using methods from environmental archaeology [57] supplemented by isotope analysis [58,59].

The so-called *Meyerhof* Švamberk consisted of several buildings used for crop processing, storage and animal husbandry. Important parts of the manor farm include staff houses and residential buildings for the temporary stay of nobles. The noble part of the manor featured a garden divided in two sections: one of them served an ornamental and representative function, while the other functioned as a utility garden for servants and subjects to grow fruits and vegetables [42].

Specifically, we aim to: (i) dendrochronologicaly date the constructions, vault infills and plant waste deposits; (ii) assess the potential of timber samples for timber provenance research in the context of the estate's economy; iii) reconstruct the spectra of utilised plants and their processing that took place directly at the Švamberk farmyard; iv) reconstruct the landscape used by the manor farmyard.

## 2 Materials and methods

**Fig. 1:**
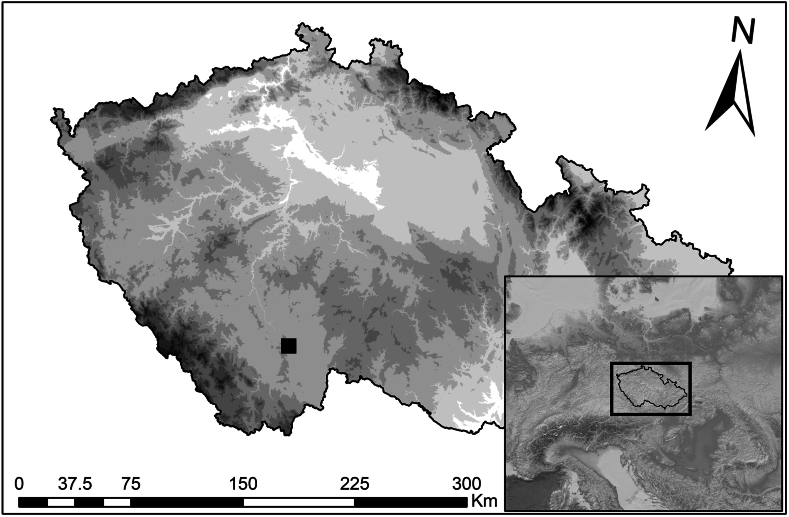
Localisation of the surveyed complex Švamberk, Czech Republic.

**Fig. 2:**
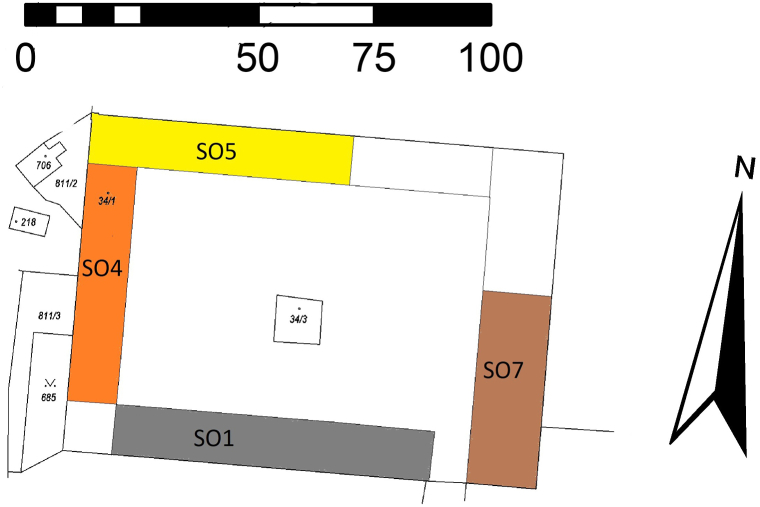
Surveyed buildings of the manor farm Švamberk.

The manor of Švamberk was located in the eastern part of the Třeboň Basin in South Bohemia, Czech Republic (WGS: 49.1180319N and 14.5926042E; Fig. 1). It was originally built by the nobles of the House of Švamberk in 1613. The construction of the wooden roofs required over a thousand trees [60]. In addition to its economic function, the manor farm also served as an occasional residence for the nobility. For this purpose, several rooms were designed on the first floor of the west entrance wing (referred to in the documentation as building SO4). This building also contained a kitchen and housing for servants (Fig. 2). During the Thirty Yearś War, the farmyard was destroyed and not rebuild until 1661 [61].

After the Thirty Years' War, the surveyed manor farm belonged to the House of Schwarzenberg, one of the most important noble families in the Habsburg Monarchy. Members of the family were active diplomats throughout Europe [62] and were also innovators in forestry and agriculture [10,14]. The Schwarzenberg dominion in Bohemia was one of the largest possessions in the Habsburg Monarchy and comprised a collection of estates and land in central, southern, and eastern Bohemia [15,63]. The economic activities of the individual estates focused on different strategies that complemented each other, creating an interconnected and diversified economic network within the dominion. The Švamberk manor farm was part of the Třeboň estate, which was primarily focused on fish farming [44,64]. The neighbouring estate of Hluboká supplied charcoal [65], while coal mines operated in central Bohemia [66]. Additionally, Švamberk was also located about 10 km from the Vltava River and approximately 15 km north of České Budějovice, making timber harvested from the mountainous regions of Šumava or Krumlov logistically well-accessible for various construction activities via floating down the Vltava [[67], [68], [69]].

Based on the available literature, we assumed [46,50,70] that the species composition of the wood used in the construction of the manor farm would change over time. We wanted to verify whether this was a result of changes in forestry practices in the area, or a change in the provenance of the raw material.

The similarity of the examined tree ring series with already dated sequences or reference master chronologies allows us to determine the age of the wooden elements, when individual buildings were constructed, and when specific artefacts were deposited [28,70]. A more detailed analysis of the wood can help us, through the mutual similarity of tree ring width sequences (see chapter 2.1), to infer the origin of the timber used in the construction of the roofs. Based on the combination of species identification and dating, we can observe how the preferences for different types of wood changed over time [46,50,70].

The ratio of isotopes ^87^Sr/^86^Sr somewhat reflects the properties of the geological substrate, suggesting that trees with a similar trend in tree ring width sequences (TRW) and strontium isotope ratios (^87^Sr/^86^Sr) may have been growing in relative proximity [59].

From the perspective of the stored archaeobotanical material, we were interested in whether the composition of the stored plants corresponds with the standard spectrum of agricultural plants represented by wheat, barley, oats, rye, oilseeds [43,52,71], or vegetables [42]. Furthermore, we examined how their composition differs compared to other sources.

The dated contexts contain archaeobotanical finds that reflect the spectrum of cultivated and gathered plants, including forest ecosystems. The combination of these methods provides insights into the character and management of the landscape [28,29,57,[72], [73], [74], [75]]. Indirectly, the by placing this information in context, we gain a clearer understanding of agricultural and forestry practices and how they have evolved during the modern era. These findings however need to be confronted and supplemented by the broader social and historical context, in this case extracted from the literature and written sources (see chapter 4.5).

### 2.1 Dating of the timbers and wood analysis

**Fig. 3:**
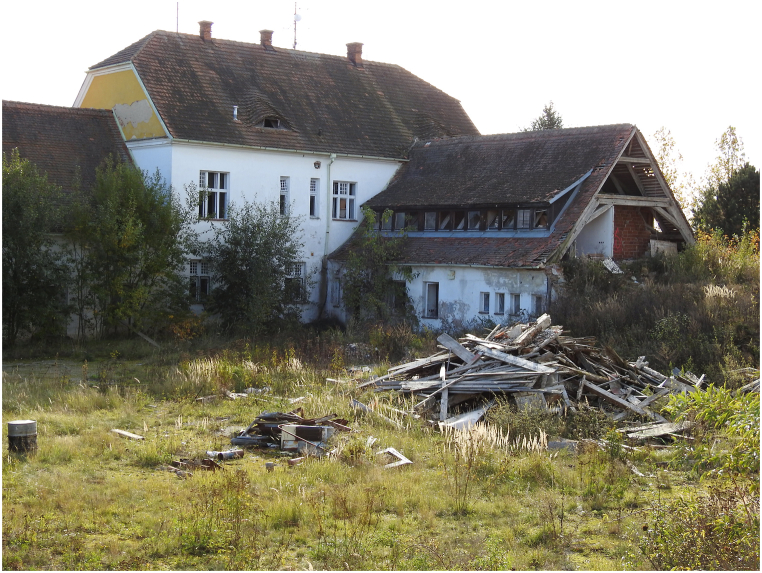
Collar roof/truss of object/building SO4 during the demolition.

The building was demolished to make way for an industrial zone, with the demolition taking place in 2019 and 2020. At the time of demolition, the Švamberk farmyard comprised several buildings of varying ages. Interdisciplinary research focused on five structures featuring Baroque elements, including the collar beam roof [76,77] Fig. 2, Fig. 3).

**Fig. 4:**
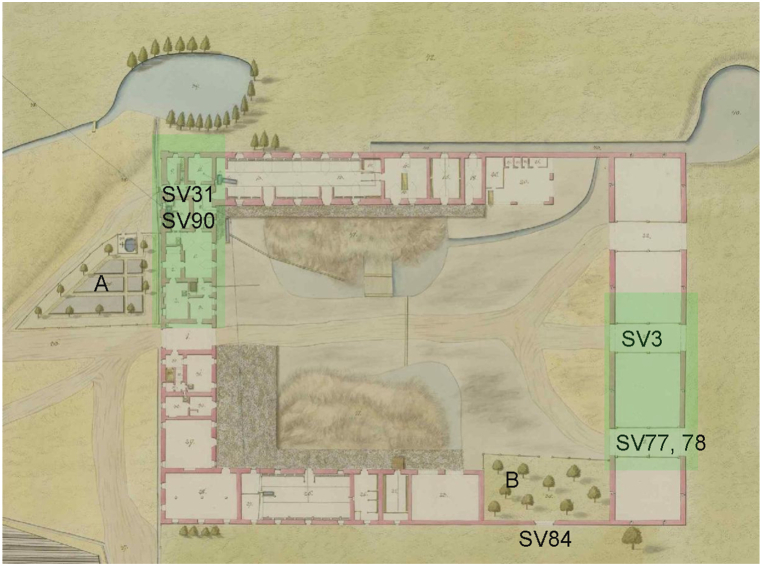
The 1811 plan of the farmyard shows A) a garden with a water tank/pot and B) a garden or orchard inside the courtyard (OA Třeboň, f. Velkostatek Třeboň, sign. IC 6 B Nro 27, inv. no. 1628, Floor plan of the Švamberk yard; sign. IC 6 B Nro 28, inv. no. 1629). Highlighted parts of the farmyard show the location of the archaeobotanical sampling. In the various parts of the complex, there are marked places of sampling of important timbers.

For dendrochronological analysis, we collected 99 samples from collar beam trusses, lintels, ceiling beams and the water pipeline.^3^ Sampling was provided using a Pressler increment borer and a chainsaw. Individual specimens were cross-sectioned and scanned at 2400 dpi using an Epson Stylus DX7450. The determination of the tree taxa used for the structural timbers was carried out through microscopic analysis of their features [78] on an Arsenal microscope SZP10032-T-N LED. The tree ring widths (TRW) were measured using Coorecorder software [79]. Presence of the waney edge^4^ was recorded provided the presence of bark allowed to recognise it.

Visual cross-dating was performed alongside statistical dating using the CDendro software by Cybis. The TRW sequences were normalised according to the method of Baillie and Pilcher [80]. Statistical cross-dating involved comparing the similarity of the TRW series by the *t*-test and correlation coefficient. Samples that did not display similarity to other sequences were cross-dated against various master chronologies [[81], [82], [83], [84], [85], [86]] or individual sequences. After cross-dating the individual samples, sequence correlation and t-value were computed with respects to the mean of the other samples (using a “leave one out” approach). The floating chronologies of each species were compared to master chronologies from central Europe and the Czech Republic [[82], [83], [84], [85],^87^,^88^].

Particular attention was given to conifers (*Abies Alba, Picea abies*, and *Pinus* sp.), which were subjects of the dendroprovenance analysis. The TRW sequences typically exhibit similar patterns in geographically close areas [89]. The location where the trees grew can be traced based on the characteristics of their annual rings and the dendroecology of each species [90,91]. Due to the relatively limited number of reference sites, the conventional method of comparing the similarity of individual samples with standard chronologies of geographically distinct areas was employed (eg. Haneca et al. [92]; Jansma et al. [93]). This method may be influenced by random similarities in TRW sequences and subjective assessment. To mitigate these issues, the method is complemented by strontium isotope ratio (^87^Sr/^86^Sr) analysis, which serves as a geochemical signature [58,59]. The ^87^Sr/^86^Sr isotope ratio in wood reflects the source composition (including a mix of more sources, e.g. bedrock, soil, soil water, and atmospheric deposition) [94]. Therefore, the isotope signal varies for trees that grew in different areas. However, assessing the diversity of strontium isotope content in the bedrock over a relatively small area can be challenging [95,96].

A total of 9 samples of hardwood (pith) were collected for strontium isotope analysis, including spruce (n = 3), pine (n = 3) and fir (n = 3). The selected samples were processed and analysed using the Thermo-Fisher Scientific Triton Plus Thermal Ionization Mass spectrometer (TIMS) at the Czech Geological Survey.

Chemical separations for Sr isotope analyses were conducted in a class ISO 7 ultra-clean laboratory at Czech Geological Survey. The procedure utilised doubly distilled acids, and 18.2MΩ·cm Milli-Q water was used throughout.

The samples were incinerated in an oven at 800 °C for 16 h. The resulting ash was then dissolved in 3 ml of concentrated HCl with a few drops of HClO_4_ at 140 °C for 48 h; after which the solution was evaporated to dryness. This was followed by three repeated dry-down steps, each using 0.5 ml of concentrated HNO_3_. Finally, the residue was completely dissolved in 4 ml of 6 M HCl.

The Sr-spec resin (Triskem Intl.) was employed for the final purification of the Sr fraction [97]. Strontium isotope analyses were performed on a Triton Plus TIMS, using a single Ta filament assembly. The external reproducibility of the obtained data was determined by repeated analyses of the SRM 987 standard, yielding a ration of ^87^Sr/^86^Sr = 0.710255 ± 08 (2SM, n = 1).

### 2.2 Archaeobotany

Vault infills and the roofs of buildings typically contain dry plant waste [28]. These features differ from commonly studied archaeological contexts, such as dry soil or wet depositions [19,98]. The main source of the archaeobotanical material for this study was dry archaeobotanical material from vault infills and the roofs.

The core of the vault infill was generally formed simultaneously with the construction of the building. However, later intrusions could also contaminate the infill, particularly during renovations. The dried plant macroremains not only allowed us to reconstruct the spectrum of plants cultivated and the layout of the farmed hinterlands but also provided insights into the processing of individual crops on the farm and information about the sediment. While written and iconographic sources recorded a variety of exotic plants present on the noble table [30], direct evidence of their cultivation remains scarce [19].

The assemblages analysed included samples from the vault infill, a sample of the daub from the ceiling, a sample taken from beneath the supporting ceiling beam (building SO4), and samples from the attic of barn (building SO7- Fig. 2; 4). Although the sample volumes were quite small, the plant material was highly concentrated.

All samples were analysed using standard methodologies [57]. Most samples were not treated, while some were separated through wet sieving. Plant macroremains were determined using identification keys and a comparative collection [72,[99], [100], [101]]. Ecological units presented in the hinterland of the farmyard were reconstructed using each species as an indicator of potential habitats. The representation of ecosystems in the vault infills waste was visualised using detrended correspondence analysis (DCA) in the Canocoo software [102]. Data underwent logarithmic transformation and detrending by segments.

The sampled plant macroremains could not be dated using radiocarbon half-life [103,104] due to the modern radiocarbon plateau, which introduces significant uncertainty in dating [105]. Instead, the age of the archaeobotanical finds had to be reconstructed through dendrochronological dating, similar to the approach used in the survey of Prague Castle [28,29].

## 3 Results

### 3.1 Quality of the dendrochronological dating

**Fig. 5:**
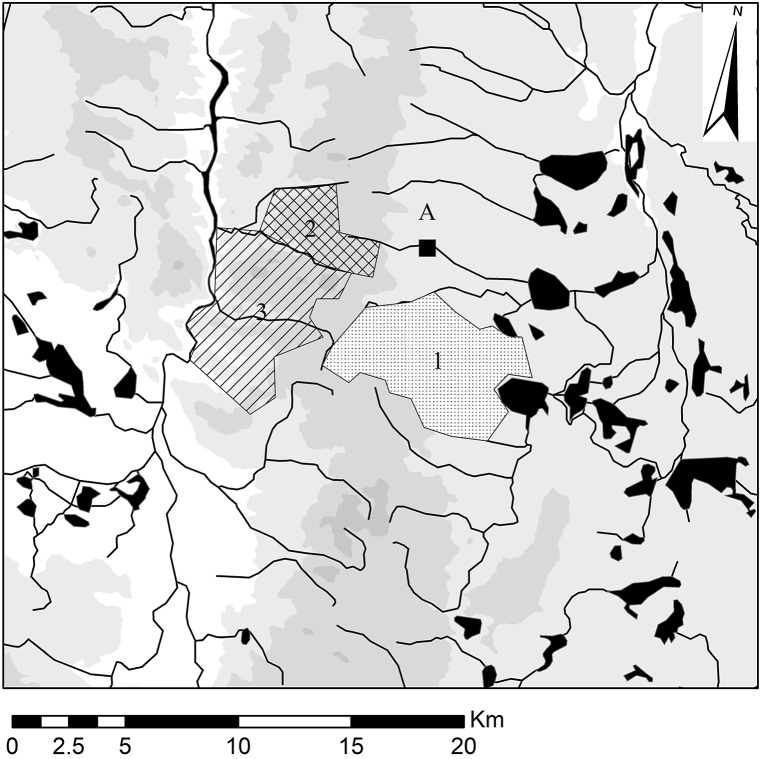
1) Close vicinity of Švamberk. Reconstructed pine forest in the 16th – 19th century [65]; 2) Reconstructed presence of spruce in the 17th century (based on Kyryanová [10]; 3) Reconstructed mixed oak-beech forest with presence of silver fir trees [65].

The collection of 99 sampled timbers contained five taxa: *Abies alba*, *Pinus* sp.*, Picea abies, Quercus* sp.*,* and *Larix decidua*. Most samples were cross-dated with other members of the same species (or with reference chronologies). While each species has distinct ecological requirements, the presence of *Abies alba*, *Pinus* sp.*, Picea abies*, and *Quercus* sp. was documented near the site during the early modern period (Fig. 5). Specimens that display low correlation with other samples were assigned positions on the best match.

Silver fir (*Abies Alba*) was identified in 51 cases. Of these, 36 wood samples displayed significant similarity, indicating that these trees were likely felled in a relatively close proximity to one another. Two additional samples (SV92, SV93) were dated according to the master chronology for Czechia created by Kyncl [83]. One sample (SV68) was dated using the South German master chronology by Becker (1970), while another sample (SV87) exhibited visual similarity to the dated sample SV86. Eleven samples could not be statistically cross-dated, so we present only the position (dating) with highest probability (Supp. material A; Supp. material B, Supp. material C). The provenance of these eleven samples remains unclear. A relatively high proportion (70.5 %) of fir samples exhibited significant similarity with the mean of the collection. Additionally mutual correlation was possible to calculate in case of 411 sequence pairs with sufficient overlap at least 20 years. Strong correlation (p∼99,9 %) was shown by 36 % of the mutually compared sequence pairs while very weak correlation was calculated in the 15 % of the pairs.

Spruce (*Picea abies*) wood was documented in 28 cases. Only 17 sample sequences showed significant similarity with the rest of the collection, suggesting a relatively uniform source area for the logs. Among these 17 samples, however, SV80 and SV81 displayed low overlap with the other sequences. Four additional samples (SV11; SV15; SV26; SV38) were cross-dated using the Šumava spruce chronology [86]. Sample SV01 was cross-dated with fir trees from Švamberk, while sample SV03 matched with chronology [84]. Sample SV51 was visually cross-dated to sample SV57 (Supp. material A; Supp. material D and Supp. material E). Only about 60.7 % of spruce samples were significantly similar with the mean, indicating a shared origin among those. A total of 96 pairs of the individual samples with sufficient overlap were available. Roughly 0.52 % of the individual sequence pairs showed strong correlation (p∼99,9 %) with each other. Low correlation showed 0,34 % of pairs.

Of the 14 identified pine (*Pinus* sp.) samples, ten sequences could be statistically cross-dated. Pinewood at the Švamberk manor was primarily used in the late18^th^ and early19^th^ centuries. The pine sequences showed a generally low correlation with each other, and also with the master chronologies (Supp. material A; Supp. material F and Supp. material G). About 71,4 % of pine samples were similar to the mean of the collection. A total of 72 pairs of the individual samples with sufficient overlap were available. Of the analysed pairs, 31 % showed a strong correlation (p∼99,9 %) while 24 % showed a weak correlation.

Three samples (all dated) were identified as oak (*Quercus* sp.) wood. Oak timbers were typically used for heavily stressed elements such as gate lintels and supporting pillars. All three oak samples date to the second half of the 17th century (Supp. material A and Supp. material H). The least commonly used taxon was larch (*Larix decidua*), which was used solely for the water pipeline (see Ciglbauer et al. [55]).

It follows that the fir trees originated from relatively similar conditions and likely from a larger area. The relatively small number of fir TRW sequences is notably different from the others. Most of the spruce samples came from a relatively small area, while the remaining portion was likely harvested from different locations. For pine samples, only a few were found that may have come from different areas, though they displayed low overall similarity.

#### 3.1.1 Strontium analysis

The differences in the ^87^Sr/^86^Sr ratios at the third decimal place suggest that the sampled trees likely originated from three distinct geological sites. The high radiogenic composition of all samples (ranging from ∼0.7187 to ∼0.7251) indicates that the bedrock from which the wood originated has ^87^Sr/^86^Sr values higher than that of atmospheric deposition (∼0.710; [94]) and is likely above ∼ 0.719, which is the least radiogenic sample in the suite.

Based on published ^87^Sr/^86^Sr data for wood, soil and rock samples from the Bohemian Massif (Czech Republic) [95,106,107], we estimate that the bedrock ^87^Sr/^86^Sr compositions for the fir and pine sites could range from ∼0,719 to 0.800. The trees may have originated from the nearby Ševětín Massif, where the granites exhibit ^87^Sr/^86^Sr values ranging from 0.717 to 0.732 [108].

Conversely, the ^87^Sr/^86^Sr of the bedrock of the spruce habitat appears to have a more radiogenic composition, with ^87^Sr/^86^Sr values potentially around 1.01, as described in the LYS locality by Ref. [107]. Here the heartwood has an ^87^Sr/^86^Sr ratio of 0.725, while the Li-granite bedrock ^87^Sr/^86^Sr registers at 1.011. Rocks with high radiogenic isotope compositions are typical of the Moldanubian section of the Bohemian Massif. Thus, accurately determining the location of the targeted forest requires a comparison of the ^87^Sr/^86^Sr compositions of wooden artefacts with those of trees from selected localities.

**Fig. 6:**
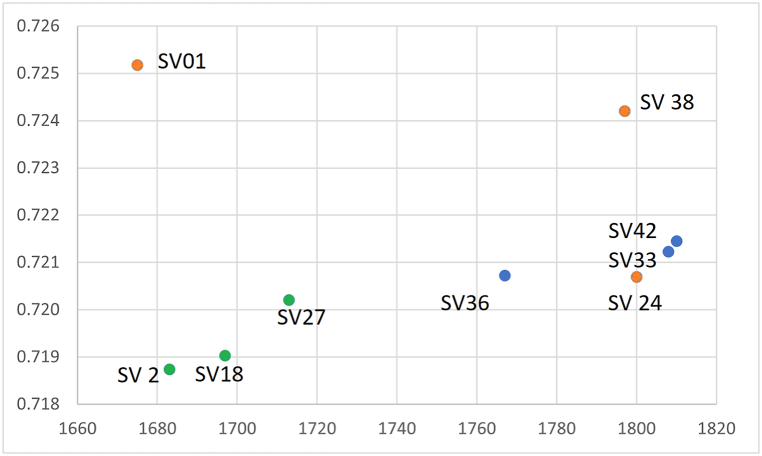
Scatterplot of the strontium isotope ratio (axis Y) according to the dating (axis X) and taxa: fir – gree; spruce – yellow; pine – blue.

All three tree species exhibited different ^87^Sr/^86^Sr isotopic compositions (Fig. 6). The most radiogenic Sr isotopic ratios were found in two spruce samples (SV1∼0.725 and SV 38∼0.724); while spruce sample SV24 (with ^87^Sr/^86^Sr ∼ 0.721) showed a similar isotopic composition to the pine samples (∼0.721). Fir samples displayed lower radiogenic Sr isotopic composition (0.719–0.720) (Supp. material I). Notably, the tree ring widths of sample SV1 (unlike the other spruce samples) exhibit significant similarity to silver fir sequences, although the ^87^Sr/^86^Sr values differ considerably. Sample SV38 which closely aligned with the Šumava master chronology [86], exhibited a high ^87^Sr/^86^Sr ratio (indicative of highly radiogenic bedrock). Sample SV24 also correlated strongly with the Sumava chronologies, yet it possesses a lower strontium ^87^Sr/^86^ ratio (Supp. material A and Supp. material I).

The Sampled pine timbers likely grew in areas with exceptionally similar bedrock ^87^Sr/^86^ ratio, suggesting they were located close to one another. In contrast, the origin of the sampled fir timbers may have been roughly near the Ševětín massif. The provenance of the spruce is more complex compared to the other wood taxa, indicating at least three distinct categories of different origins.

### 3.2 Dating of the vault infills and contribution of dendrochronology to the building history

#### 3.2.1 Dating of the deposits from the residential building (SO4 northern part)

The dendrochronological analysis of joists (a ceiling type made from split trunks and clay, originally called *poval*) and wooden floors allowed us to establish a terminus post quem (the vault infills had been deposited before the joist ceiling was constructed). Similar approach was employed in the studies by Kosňovská [28]; Beneš et al. [29].

**Fig. 7:**
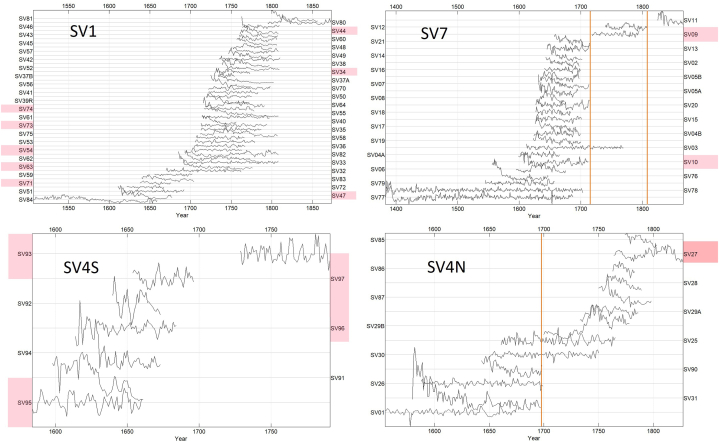
Plot of the TRW sequences from the individual buildings. Orange line show the presence of a waney edge. Highlighted sequences could not be dated with confidence. Each series is detrended by subtracting the series mean.

Analysis of the joist half-beams (*poval*) from the northern wing of the frontal building indicates that joist ceiling was constructed around the year 1698 (Sample SV31 was felled in the year 1697; sample SV90 was felled in 1697). The wall beam (Sample SV01) from the same area was made after 1675, while the floor above the joist ceiling was dated to after 1786. Due to heavy treatment, we do not have the waney edge measurement for the plank, but it was likely to felled around 1810. The tie beam and frame (samples SV85, SV87) were dated (no waney edges) to the years 1798–1803 (Supp. material A; and Fig. 7).

**Fig. 8:**
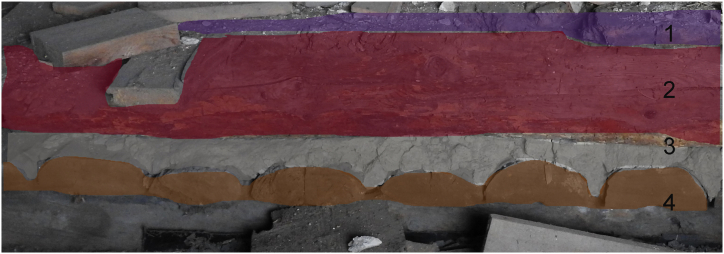
Section of the wooden floors and joist ceiling above the vault infills 1. Layer of the floor (after 1786) 2. Tie beam and the frame above the joist ceiling (after 1798 to 1803) 3. Daub layer of the joist ceiling 4. Joist (*Poval*) wooden construction (1697/8).

Regarding the northern wing (building SO4 north), repairs to the roof trusses and floors from the early 19th century likely did not impact the vault infill, as the layers of the joist ceiling above the vault infills remained intact during excavation (see Fig. 8). Artefacts deposited alongside the plant waste are classified as mid-17th century based on typological dating [109]. Although the archaeobotanical material from the vault infills was likely deposited at the end of the 17th century, some portions of the assemblage might be older (Supp. material J). The available written sources do not document the cultivation of certain crops found in the deposits [110].

#### 3.2.2 Dating of the deposits from building SO4 (southern part of residential building)

Samples from the southern and central sections of the residential building (SO4 south; Fig 2; 4) suggest that the joist ceiling was constructed in the multiple phases: first after the estate was acquired by the House of Schwarzenberg in 1661 (samples SV91; SV95) and then after 1673 (samples SV92, SV94). Sample SV93 can be dated to either 1676 or 1791; however, neither date reaches convincing values of the r^2^ or t-value (Supp. material A).

The core of the archaeobotanical assemblage found in the southern wing of buildings SO4 is likely dated to the 17th century. It is probable that the archaeobotanical material was deposited during younger periods (see the dating of the timbers Fig. 6; Supp. material A and Supp. material J).

#### 3.2.3 Dating of the deposits from storage barns (building SO7)

Organic sediment samples from the SO7 building can be dated based on the historical building survey (HBS) and dendrochronology. The HBS indicates that two brick, cross-passage barns in the eastern wing of the yard were established as early as 1711, with new storage barns built in 1715 [54,61]. The spruce lintel of the gateway of the SO7 storage building was made after 1769. The wooden columns supporting the tie beams were dated to after 1647 (SV76) and 1657 (SV79), although the waney edges of these samples are not present.

The dates from the trusses range from 1665 to 1875, with the majority of the dates occurring in the late 17th and early 18th centuries (Fig. 7). Therefore, the core of the building dates back to the first rebuilding period following the Thirty Years’ War. The rafters were repaired subsequently. The dates from the samples containing the waney edge indicate periods of truss repairs and possible reconstructions. One waney edge date (SV13), along with other samples (SV21, SV7, SV8, SV10), aligns with the recorded construction date of the brick barn in 1715. Additionally, another repair documented by a sample with a waney edge occurred in 1807 (SV12).

Samples for archaeobotanical analysis were collected from the narrow space between the crown of the wall and the wall beam. Their age is uncertain; it can be estimated to fall within the late 17th to mid-19th century range. It is possible that this plant material was deposited beneath the layers during one of the building's reconstructions (Supp. material J).

#### 3.2.4 Dating of the other buildings

Although no archaeobotanical sample was taken from the SO1 building, the dating of the timbers provides valuable data about the building's history. This structure was used as stables and a cattle stall. The oldest timber feature discovered in the building is the gate lintel (SV84), which was originally part of the wall behind the garden (Fig. 4). The date of the last tree ring is 1658, although the waney edge was not present. The majority (middle 80 % quantile) of the wooden samples were dated to the period between 1750 and 1810. The youngest timber was dated to 1873. The production building, SO5, was characterised by only three samples, all of which were dated to the end of the 18th century (Supp. material A).

All samples with statistically and visually robust dating results (n = 78) were divided into two roughly equal intervals: before and after 1750. In the earlier period, fir made up 76 % of the collection, but after 1750, this percentage dropped to 31 %. The proportion of spruce wood increased from 12 % to 49 % in the later period. Pine wood was minimally represented in the earlier period at 3 %, but after 1750, it accounted for 20 % of the examined samples. Oak was recorded only in the period before 1750, with only three specimens.

### 3.3 Plant macroremains

**Fig. 9:**
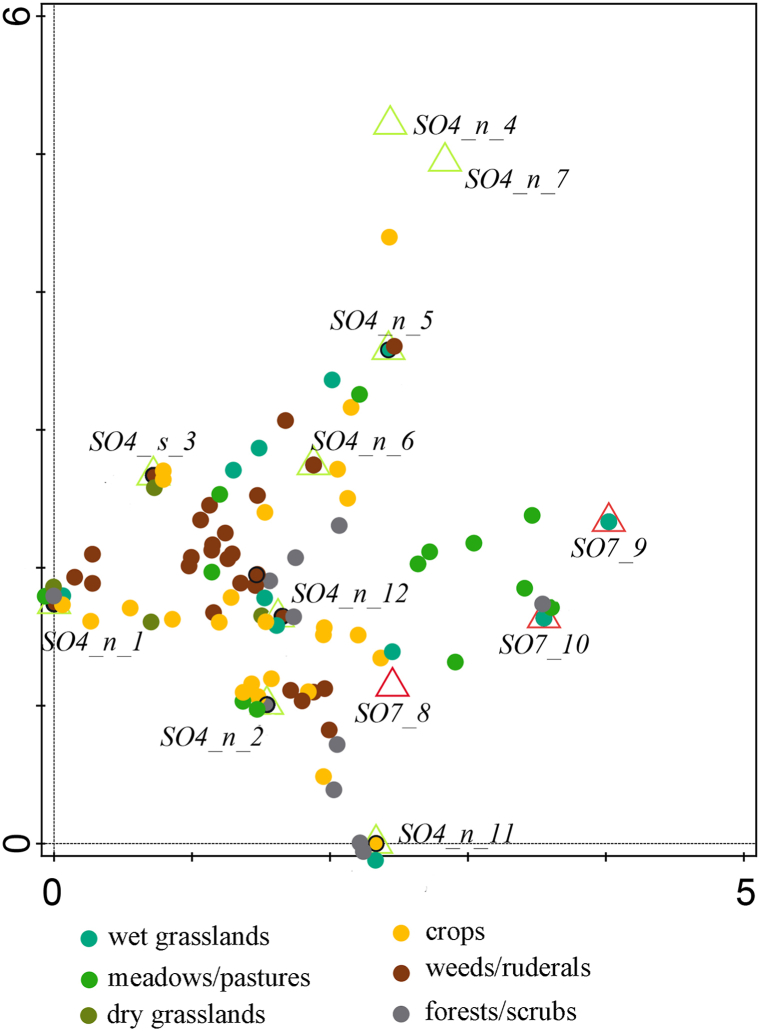
Visualisation DCA results of the habitats represented in buildings. First axis explains 21,62; both axis explain 33,4 Samples from vault infills and roof of residential buildings (SO4) - green triangles; samples from roofs of storage barns (SO7) - red triangles. Species are marked by dots, ecology of individual species is distinguished by colour.

**Fig. 10:**
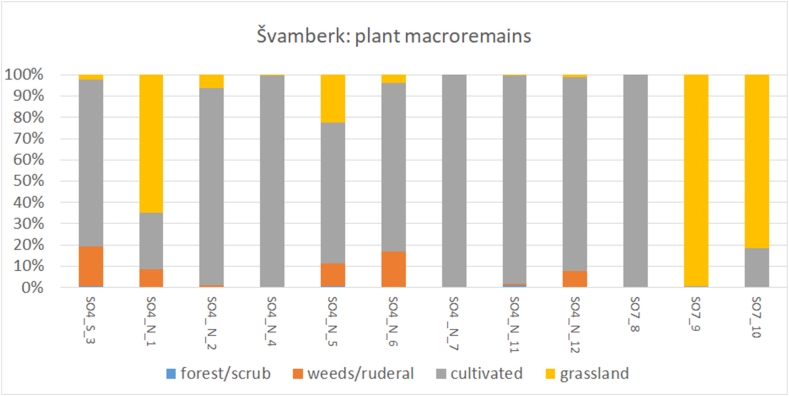
Plant material of various habitats characteristic for the different samples.

The collection of plant remains recovered from the roofs of the manor farmyard Švamberk is notably numerous, amounting to 81, 892 individual specimens (Supp. material J). This assemblage was also exceptionally diverse, with approximately 120 different plant taxa recorded. The concentration of plant material per litre of sediment was extraordinarily high, with minimal presence of other admixtures. Only sample 3 contained dust and sample 6 included fragments of daub. Sample 7 consisted, on the other hand, solely of daub. The assemblages from individual buildings vary considerably, yet many plant species are recurrent across the samples, reflecting the shared farmstead environment but differing deposition activities. For instance, the samples from the southern part of building SO4 contained cultivated plant remains, while the samples from building SO7 predominantly featured grassland plant remains (Fig. 9, Fig. 10.).

**Fig. 11:**
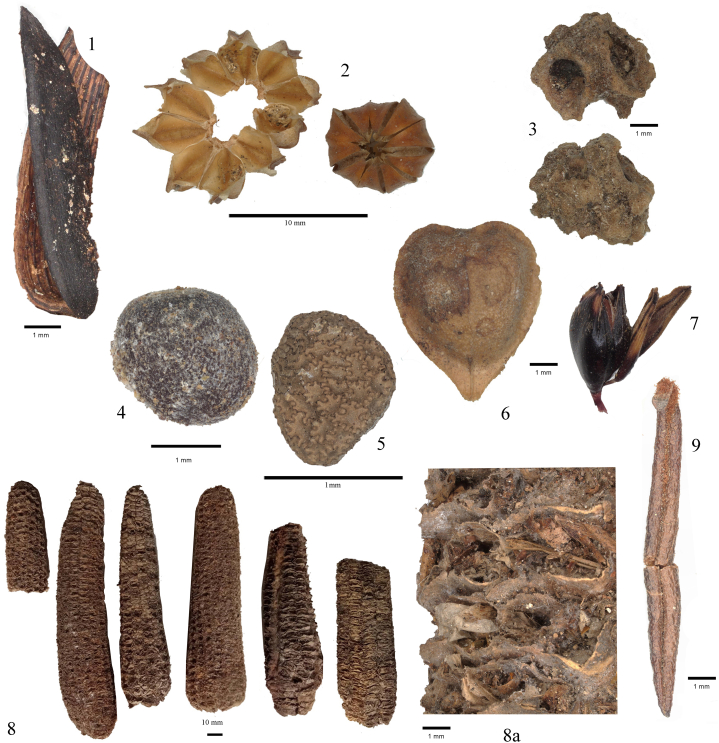
Important macroremains present in the dry sediments of the Farmyard. 1 *Helianthus anuus;* 2 *Linum ussitatissimum;* 3 *Beta vulgaris;* 4 *Brassica Oleracea;* 5 *Nicotiniana rustica;* 6 *Camelina alyssum* subsp. *alyssum;* 7 *Sorghum bicolor;* 8 *Zea mays;* 9 *Ledum palustere.*

In the northern residential building (SO4 N with vault dated 1698), dry grassland plant remains made up a significant portion of the assemblage. Plants from cultivated fields and ruderal habitats also appeared in substantial numbers. In contrast, meadow, pasture, wet grassland and woody plants were less common. Notably, the assemblage contained a large number of neophytes-plant taxa introduced to Europe during the early modern period, primarily from the Americas (Fig. 11). The vault infills of the northern residential building (SO4N) included macroremains of these species. Among the most significant finds was *Nicotiana rustica* seed, along with the first archaeobotanical evidence of *Zea mays* cultivation in central Europe. *Zea mays,* originally domesticated in Central America (Stitzer and Ross‐Ibarra, 2018), was present in large quantities. Additionally, the vaults of SO4N building contained numerous remains of the African *Sorgum bicolor* and *Parthenocissus*, a genus native to Asia, namely to the Himalayas.

In the southern part of the frontal building (SO4 south), remains of cultivated plants are primarily represented by a collection of field and ruderal plants; other plant categories are sparsely documented. Sample 1 from the vault infill contained macroremains of *Helianthus annuus* imported from North America. Additionally, the seed of *Platycladus orientalis* was present in this sample. This species, originally ornamental, with a natural range in China and far East, represents another example of modern-age botanical import.

In SO7, the plants of meadows and pastures are mostly accompanied by macroremains of cultivated plants (Fig. 9, Fig. 10). The presence of the *Zea mays* in the storage barn (SO7) was confirmed by the discovery of maize bract remains. A summary of the overall results is available in Supp. material J.

## 4 Discussion

### 4.1 Timber provenance

An intriguing aspect of the economy of the Švamberk estate is the logging and timber transportation. The temporal distribution of the taxa in the dated samples shows noticeable shifts in the types of timber used for farm repairs and reconstructions. In the 17th and 18th centuries, a high abundance of silver fir timbers was recorded; however, after the 1750s, a significant portion of trusses was made from spruce and pine. The increased use of spruce and pine timber in the late 18th century is a phenomenon observed across broader regions [46,50,87,111]. However, in South Bohemia, this trend shows significant differences between observed sites [46]. Common interpretations include developments in forest management, climatic variations, or advancements in timber transportation technology [46,50,73].

In most cases, it can be assumed that local wood was used [111], although long-distance imports are also documented [10,67,69,112]. The high proportion of utilised fir timbers likely reflects its abundance in the area surrounding the manor farmyard. About 3 km southwest of the farm, the Ševětín Upland, at an elevation of around 500 m a.s.l., offered an ideal environment for silver fir growth. Research of charcoal kilns in various locations of the Ševětín Uplands also confirms abundant presence of silver fir in local forests, which were largely composed of oak and beech in the 15th - 18th centuries [65]; Fig. 5). Presence of the fir and spruce needles in the vault infills suggest that the some fir and spruce trees were cut and treated locally.

It is likely, that most of the fir trees originated from a geographically distinct area, potentially in the Ševětín Uplands. The uniformity in ^87^Sr/^86^Sr ratios measured in the wood of fir trees supports this hypothesis. However, fir timbers from the southern wing of the building SO4 do not match the fir sequences from the rest of the farmyard and their origin is likely to be different. Although the proportion of fir wood used in roofs decreased, it is evident that fir still constituted a high proportion of the surrounding forests in the 19th century.

In contrast, a significant increasing trend was observed in the use of pine wood, likely related to changes in vegetation cover due to intensive human activities. Based on the ^87^Sr/^86^Sr values, the pines likely originated from a single area, possibly the Třeboň Basin, where wet conditions and acidic sandy soils provide ecological optimum for pine growth [113].

Antracological survey [65] indicates a significant presence of pine in areas heavily impacted by human activity, where the natural succession from oak to pine forests occurred during the Middle Ages and Modern period (Fig. 5). The increasing proportion of pine wood in the Švamberk collection can likely be attributed to changes in vegetation cover caused by human activities, forest grazing by nearby sheepfolds, and logging for charcoal production in the Hluboká estate, 2 km away. These activities depleted nutrients and caused succession favouring pines which grow on poor soils and in extreme habitats.

Spruce wood replaced the fir as dominant tree in the assemblage after 1750. Spruce, with its shallow root system, tends to thrive in stable and wet environments, particularly in mountainous areas [113,114]. Nevertheless, spruce has grown spontaneously in lowland areas, especially in wet habitats [115]. Evidence of spruce harvesting within 5 Km (450–550 m a.s.l.) radius of the site, dates to as early as 1693 (Fig. 5). The spruce wood was probably intended for export, given its high market value [10], local fir wood was preferably used for most constructions at the manor farm. Similar to the oak wood, more prominent features, such as the wall beam (SV01) and the lintel (SV03), were made of spruce.

**Fig. 12:**
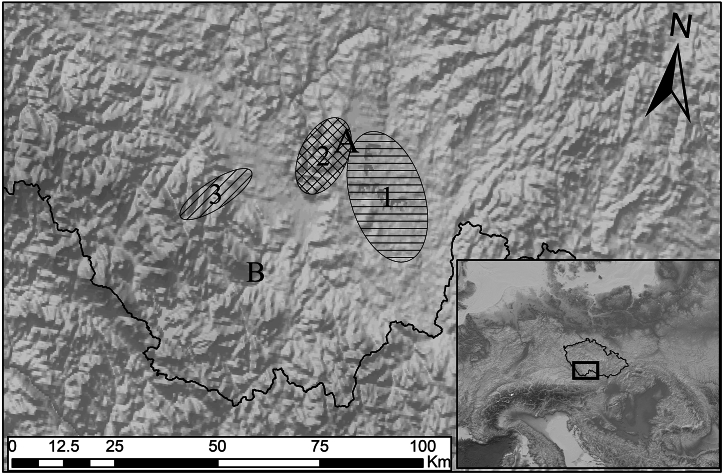
Areas of high significance for the timber provenance analysis. A: Švamberk Manor, B: Český Krumlov acquired in 1719 by prince František A. of Schwerzenberg, 1 Třeboň basin, 2 Ševětín Highland, 3 Šumava Mountains represented by the master chronology of Kolář [86].

**Fig. 13:**
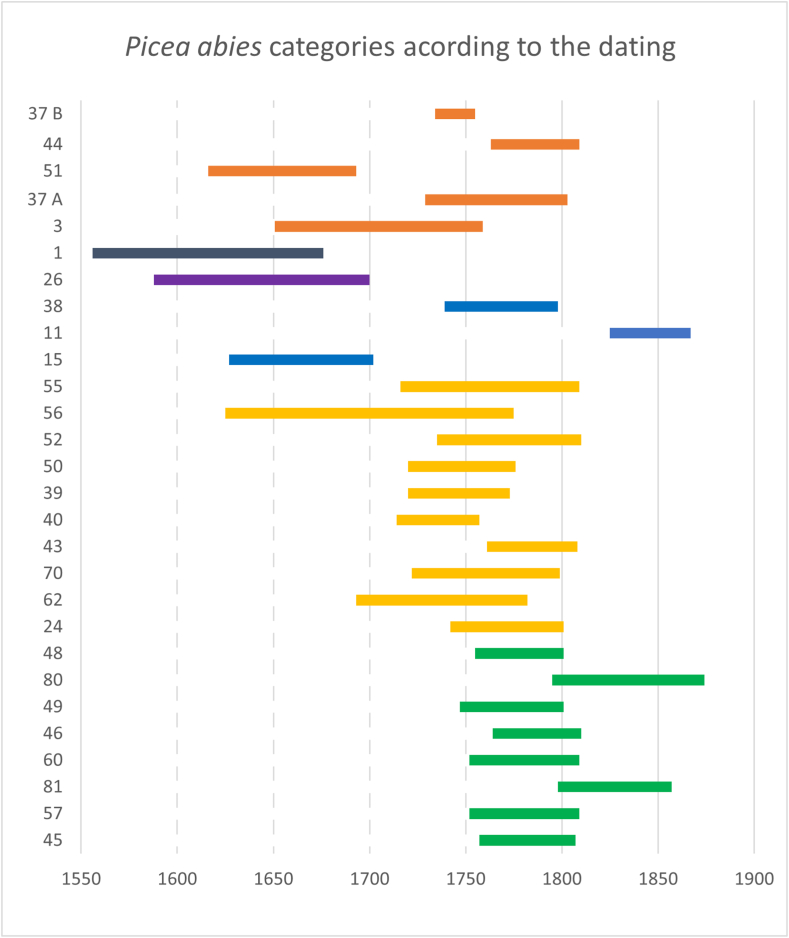
Plot of spruce chronologies. Sorted according to the properties of the TRW sequences. Orange: Samples with low correlation with used masters Yellow: sequences that match each other (they possibly come from the same area, but we don't know exact place) Blue and purple: sequences similar only to the reference chronology from Šumava-probably originating from Šumava foothills Dark blue: sequences matching fir trees-probably originating from the Ševětín Uplands Green: sequence matching the Šumava chronologies but also the mean of spruce sequences in the assemblage.

The Schwarzenberg dominion encompassed much of southern Bohemia, including mountainous areas where spruce forests naturally thrive [33] or replaced the fir as consequence of human activity [116]. In 1719, the Schwarzenberg acquired the Krumlov estate, and in 1799 the Prášily estate in mountain regions, therefore spruce wood became more accessible [112]. The provenance of spruce wood, however, proves to be particularly complex. The ^87^Sr/^86^Sr isotope values in spruce samples indicate diverse origins (Fig. 6). The results of strontium isotope measurements reveal that the spruce wood used in the construction of the Švamberk farmstead, at least in two cases, grew on different bedrock than that of fir and pine (Fig. 4; 5 and 6). Although most spruce timbers show relatively good cross-correlation, suggesting they were likely transported from a single area, the exact location remains uncertain (Supp. material E). A portion of spruce timbers shows a strong correlation with Šumava mountain chronologies, suggesting they may have originated from the Šumava mountains or their foothills). Meanwhile, certain spruce trees (represented by the sample SV1) grew possibly in the Ševětín Upland, though on different bedrock from that of silver fir (Fig. 12, Fig. 13).

The wood from the Krumlov or Šumava foothills estate could have been most easily transported via the Vltava River and used in the construction of Švamberk. Meanwhile, timber from the Prášily was floated to Prague by a different route [112]. The timber was used not only for the Schwarzenberg's construction activities but also for trade. Timber of various species, including spruce, was transported from both Hluboká and Český Krumlov by the Vltava River. The gradual addition of wood to the rafts from various places along the trade routes is also documented [10,13,117].

### 4.2 Vault infills

The waste left after processing the plants at Švamberk provided information about various aspects of the farm, which was involved in their cultivation. The same plants are documented in the environment of urban pits in the form of kitchen waste [19]. The sediment from the vault infills of the farmyard, which consisted of dry plant waste, provide direct evidence of plant cultivation (including exotic plants). Vault infills also provide information about the weediness of fields and gardens/beds, and the processing methods of individual crops.

This type of archaeobotanical deposit has so far attracted very limited research interest [28,29,68]. So far surveyed vault infills describe a very different social context compared to the deposits discovered at the Švamberk manor farm. While residential city houses and palaces contain plant macroremains, these are primarily from plants which were consumed rather than produced at the site. As a result, such urban features often include a large proportion of luxury imports [28]. Research into production centres, such as rural houses and manor agricultural buildings, could greatly improve our understanding of early and high modern agricultural economies.

### 4.3 Reconstruction of the grown plant spectra and plant processing

In the roof constructions at Švamberk (SO4N, SO4S and SO7), macroremains of a wide spectrum of cultivated economic plants were discovered (Supplementary material J). In most cases, the findings consisted of production waste, such as husks, empty capsules, crushed seeds, etc. The most common cereal grain found was oat (*Avena sativa).* A large proportion of the remains were empty husks, indicating that the oat was prepared as human food rather than as fodder for animals (as the husks were separated). Additionally, husks with awns and the awns of the *Avena strigosa* (bristle oat) were also found. The bristle oat is a species that was sometimes cultivated in areas where common oat did not thrive. In climatically suitable regions, it appeared as an unintentional admixture. Although it is a species native to Europe, it is now regionally extinct in the Czech Republic [118]. Barley (*Hordeum vulgare* var. *vulgare*) was documented in the form of husks or spikelets, with only a few hulls found. Similarly to the case of oats, barley processing waste was present in the vaults of the investigated features. Wheat (*Triticum aestivum*) was documented only in the form of a few spikelets and husks; rye (*Secale cereale*) was found solely as husks.

Surprisingly, finds of the processed maize ears (*Zea mays*) were also discovered. Maize originated in the Americas and reached Europe in the 16th century [20,[119], [120], [121]]. It is referenced in written and iconographic sources, maize starch has been also detected, however, direct archaeobotanical findings has been lacking until now [31]. Some sources suggest that maize may have entered central Europe from the eastern Mediterranean region via the Balkans and Anatolia. Portuguese trade played a more significant role in the spread of the neophytes than the Spanish trade. Through Portuguese colonies in Indonesia, commodities and ideas spread into the Indies and Middle East. By 1536, a significant portion of crops produced in the Ottoman Empire included maize and other plants from the New World [122]. Surveys conducted by Habsburg officials after wars with the Ottomans (1722) revealed that 31 % of cereal production in the Habsburg-aligned Kingdom of Serbia consisted of maize [123].

It is likely that maize and other crops were imported into central Europe through Ottoman territories in the Balkans, either via trade or from reconquered lands. Another possibility might have been the transport of seed through the Habsburg Netherlands, where, for example in the 17th century Prince Jan Adolf of Schwarzenberg was active at the court of the Dutch governor Leopold Wilhelm of Habsburg [62].

Another non-indigenous crop is *Sorghum bicolor*, which originates from Africa [124] and is highly drought-tolerant. However, there is no available Information on sorghum cultivation in early modern times in central Europe, nor have any known archaeobotanical finds been discovered. In contrast, *Fagopyrum aesculentum* (buckwheat) has been cultivated in central Europe since the Middle Ages and it was a common crop.

A number of oilseeds have also been discovered, predominantly *Linum usitatissimum*. A large quantity of seeds was found, many split lengthwise, and in some cases, bigger clumps of seeds were pressed together. It can be assumed that these seeds are waste from oil pressing carried out in oil mills [125]. In addition to the seeds, empty capsules and their fragments were present, further suggesting waste from linseed processing. In the early modern period, linseed oil was primarily used for technological purposes and as an additive in horse and cattle feed [71].

Another plant, found in the deposits at Švamberk, potentially used for oil and textile production, was *Canabis sativa*, though unlike *Linum usitatissimum*, it was found in small quantities. Additionally, *Sinapis alba* (white mustard), another oilseed plant which was used as a spice, was documented [126]. Of particular note is the discovery of *Helianthus annuus* (sunflower) seed coats, a plant native to North America. Sunflowers reached Europe in the early 16th century, initially cultivated as ornamental flowers, and by the second half of the 18th century, at the latest, they were already used as oilseeds [127].

Among the cultivated vegetables, root crops such as *Beta vulgaris*, *Daucus carota*, and *Apium graveolens* were recorded. A few peels document the cultivation of *Allium cepa* and *Allium sativum.* Seeds of *Brassica oleracea* were found in large quantities. While the specific variant of *Brassica oleracea* could not be identified, cabbage, cauliflower, broccoli and Brussels sprouts are all considered possibilities. Written sources mention the cultivation of two types of cabbage: blue or red, as well as curly and barrel cabbage, which could be green, white, or red, along with kohlrabi [56,110,126].

The large number of *Sanguisorba minor* fruits, which predominated in Sample 1, indicates either systematic collection or intentional cultivation. According to Mattioli [110], *Sanguisorba minor* can be used as either a leafy vegetable (like spinach) or as a medicinal herb for treating dysentery, bleeding wounds, ulcers, or plague fever.

The fruits of exotic plants found in the sample under the lintel in the residential building in the southern part of the courtyard may be linked to the ornamental garden belonging to the nobleman's house. The fruits of cf. *Platycladus orientalis* and *Parthenocissus* cf. *tricupsidata* could indicate the importation of seeds/plants from Asia. According to written records, *Platycladus orientalis* was introduced to Europe in 1737, with the first record of its planting in Bohemia dating to 1776 [128,129], while *Parthenocissus tricupsidata* was cultivated in Bohemia only in the late 18th century; Čulíková [19] also mentions the discovery of a seed fragment probably from *Parthenocissus inserta*, which is, native to North America.

There is limited evidence of orchards and fruit tree cultivation. Documented species include *Prunus domestica* and *Prunus insititia*, *Malus domestica* and *Pyrus communis*. Fruit trees may have grown directly within the yard, particularly in its southeastern part, as indicated on a plan from 1811 (Fig. 4, marked B), or in the backyard, such as northwest of the sheepfold near the farmyard, where trees along the meadows/pastures were depicted in the second military survey.

An important find was a seed belonging to the tobacco species *Nicotiana rustica.* Originating in the Americas, the plant rather quickly made its way into Europe after its discovery at the end of the 15th century. Seeds of the tobacco plant reached the Iberian Peninsula in the year of 1518, and since then, they were frequently planted in ornamental gardens. In the 16th century, tobacco was also valued for its medical properties. During the 17th century, tobacco cultivation became common in the United Provinces, France and the Italian states. In German states and central Europe, the spread of tobacco smoking was recorded since the Thirty Years' War [130] largely due to international mercenary armies, and the plant became an important commodity. In the second half of the 17th century, tobacco was grown sporadically in Bohemia [131]. The earliest tobacco pipes found in central Europe date back to the 17th century [132,133].

Archaeobotanical finds of the tobacco seed are rare. The oldest discovered tobacco seed in the central Europe was found in a cesspit at Prague Castle. It is dated to the period from the late 16th to mid-17th centuries. Its presence may be linked to the activities of Spanish diplomats at the end of the 16th century or to the St. Anthony infirmary [19,130]. The tobacco seed found in the vault infills of the SO4 (north) could have been deposited in 1697/1698 (see results), possibly indicating tobacco cultivation for the needs of the nobility. Compared to cultivated tobacco (*N. tabacum*), *N. rustica* was primarily used for medicinal purposes as it was less suitable for smoking [29].

### 4.4 The landscape surrounding the manor farm

The spectrum of documented plants allows for the construction of an open agricultural landscape with various types of biotopes that were intensively used by humans. Pollen analysis of the sediment from the coin hoard at the church in Bošilec (approximately 5 km away [134] presents a similar view of the 17th century landscape. The botanical data confirms the presence of fields dominated by cereals, numerous oil plants and relatively few legumes. The cultivation of vegetables is uncertain– some may have been grown in backyard gardens, while others were likely cultivated in the fields. The fields were infested with a wide range of weed species, but no single species predominated. This suggests intensive agricultural activity and a diverse mosaic landscape restored around the manor farmyard after the Thirty Years' War (Fig. 9, Fig. 10).

The number of weed species also corresponds to the variety of crops grown, each thriving in specific conditions. Winter crop weeds included for example *Agrostemma githago*, *Centaurea cyanus* and *Lithospermum arvense*. A characteristic weed is *Raphanum raphanistrum*, which was virtually impossible to remove from the grain, due to its large fruits.

Seeds of *Anthemis arvensis*, common bracken (Fallopia *convolvulus*), *Chenopodium album*, *Galeopsis tetrahit/bifida*, *Persicaria lapathifolia*, *Valerianella dentata*, *Polygonum avicular*, *Rumex acetosella* and *Scleranthus annuus* were present in significant quantities.

The landscape was structured among smaller fields, pastures and meadows where perennial forage crops grew – primarily *Poa pratensis*, *Festuca ovina*, *Trifolium pratense, T. arvense, T. repens,* and *Vicia hirta.* The area surrounding the farmyard was rather humid, as evidenced by the presence of wet grass species such as *Carex* cf. *pallescens*, *Scirpus sylvaticus*, *Ranunculus acris*, *R. repens*, and *Rumex acetosa*. Waterlogged fields or water banks are characterised by *Equisetum palustre*, and exposed shores or pond bottoms by the *Bidens radiata*. The leaves of *Ledum palustre* indicate the exploitation of resources from peat bogs and the *Lycopodium clavatum* likely grew in coniferous forests, heaths and pastures.

The use of resources from the forest environment, forest margins, and shrubs is evidenced by the findings of the remains of potentially collected fruits and nuts belonging to black elderberry *(Sambucus nigra*), hawthorn *(Crateagus*), rosehip (*Rosa canina*) and hazelnuts (*Corylus avellana*). Seeds, fruits, and leaves of trees also indicate the exploitation of forest resources, including sycamore maple *(Acer pseudoplatanus*), silver birch (*Betula bendula*), oak (*Quercus* sp.), large-leaved and heart-leaved linden *(Tillia platyphyllos, T. cordata*) and silver fir (*Abies alba*).

The historical landscape was not designed randomly but rather to support sustainable economic production in varied conditions [135] (In the case of Švamberk the surrounding wetlands and woodlands).

Various habitats documented in the hinterland of Švamberk were conserved and effectively utilised. Landscape division into different habitats such as grasslands (meadows, fields); shrubbery (forest fringe, hedges), woodlands (forests, orchards), water bodies, and wetlands (ponds, streams, rivers) helped maintain biodiversity [8,9,136]. Ponds systems were effective in management of groundwater, in contrast to drainage systems, which could cause unwanted hydrological changes leading to severe droughts [137]. Tree alleys, hedges, and orchards protected against soil erosion, while livestock breeding supplied fields with biomass, helping to prevent soil depletion [138]. Changes in the structure of the rural landscape could potentially be detrimental to the environment leading to droughts, depletion of the fertile soil, and destruction of historical monuments and cultural heritage [5,8,9,136].

Destruction of the surveyed farmyard, represents case of trends striving to preserve environmental sustainability that often lead to the relocation of industrial centres from urbanized areas to less developed agricultural landscapes [see 135]. The consequences include the destruction of historical landscapes and unique ecosystem structures, resulting in a decrease in biodiversity and the usability of landscapes for agriculture.

Nowadays landscape features are primarily used and restored to improve the hydrological condition, enhance water retention capacity, and to slow erosion [136,137]. However, in practice, this is often done carelessly and without preserving elements of cultural heritage. Buildings such as farms, chapels, and groves are part of the cultural heritage, and may also contain archaeological material, including artefacts, seeds, architectural and wooden features, which have the potential to complement written sources.

In addition to the destruction of historic buildings, waterlogged sediments that preserve pollen grains and archaeological features with present macroremains are often destroyed. These archives carry crucial information about landscape transformations in the context of human settlement [138,139]. They can thus significantly contribute to the understanding of landscape development, which can be used for more effective utilization and revitalization. An ideal approach could involve palaeoecological and archaeological research of the area of interest, based on which historical landscape elements would be restored or revitalized to maximize their functionality. This would lead to the preservation of information, optimization of landscape functions, and the conservation of biodiversity.

### 4.5 Findings from Švamberk in the larger scope

The core of the archaeobotanical collection likely reflects waste from agricultural production, food processing, and everyday life at the manor farmyard of Švamberk. The waste found in building SO4 was probably produced continuously during the second half of the 17th century and subsequently deposited in the vault infills. These vault infills (dated 1697/8) might reflect the economy of the manor (via waste remains) farm during the reign of Prince Ferdinand of Schwarzenberg (reigned 1683–1703) [15] and his Father Jan Adolf (reigned 1660–1683) [37]. Written sources mention the rebuilding and constructions of various manor buildings, corresponding with the dendrochronological dating of the wooden structures. Comparison of our results with the studies aimed at the broader picture of the Schwarzenberg estates (see Ref. [10]) allows us to propose explanations for the phenomena observed in the archaeological material.

Developments such as the introduction of new species into crops and changes in timber provenance may be linked to innovations and structural changes at the Schwarzenberg dominion after the Thirty Years' War, particularly at the end of the 17th century and during the 18th century. These changes were also connected to forestry and agriculture [10] and carefully coordinated by the governor and the burgrave with the estate owner [60].

Linking dendrochronological and botanical data to the management system and the economic doctrines of the studied period might provide basis for the possible explanation of the unusually high presence of the neophyte taxa. The Schwarzenbergs naturally sought self-sufficiency on their estates, aiming to minimize imports from abroad, neighbouring estates or purchases from peasants. A key focus was self-sufficiency in barley production and beer brewing [33]. The attempt to cultivate unusual crops directly on the Třeboň estate in central Europe during the 17th century may have been more than a mere agricultural experiment; in the context of the time, it was likely a well-considered business plan by the economic elite.^5^ The reign of Prince Fratišek Adam Schwarzenberg (reigned 1703–1732) and his son (1732–1782) marked another period of significant change and frequent reconstructions [140]. For example, the object SO7^6^ (storage barn) was built in the year 1715 [54]. The reign of Prince Fratišek Adam Schwarzenberg (reigned 1703–1732) and his son (1732–1782) marked another period of significant change and frequent reconstructions [140]. For example, the object SO7^7^ (storage barn) was built in the year 1715 [54].

Moreover, the economy of the farm did not exist in a vacuum. Discoveries and establishment of international global trade caused introduction of exotic crops into European society. Changes in the global patterns likely influenced availability of various goods local customs and the economic decisions of the nobility. At the beginning of the 18th century, during the reign of František Adam Schwarzenberg, a significant change occurred in the Atlantic trade. The Habsburg defeat in the War of the Spanish Succession brought Spain, which had been the Habsburg gateway to the Atlantic trade, under the influence of France. Consequently, the French kingdom became the hegemon controlling the import of overseas goods into Europe. However, these events did not have a major impact on trade routes, and overseas goods did not become unavailable. Nevertheless, the lost war may have affected perceptions of overseas trade as being unstable, as it relied on many various factors [22]. This event could have strengthened the incentive to produce various exotic crops.

The production of a wide range of new crops could have helped spread the risk of crop failure over different species with varying ecological requirements, such as maize, millet, and sorghum, which are less sensitive to drought. In contrast, oats and barley are drought-sensitive crops that require rather wetter and cooler conditions [141,142]. Most of these crops would be used directly for production or stored. Barley was a key raw material for food production and was used in brewing, while oats served as fodder for horses. Notably, the documented cultivation of tobacco, if produced on the homestead, could have been intended for a wider market as a consumer good.

The pattern of agriculture until the modern era demonstrates a more or less continuous development. At the Švamberk manor farm, a transition from conservative agriculture to a modern system can be observed. The utilization of economically important crops was complemented by a variety of new and exotic species, which were likely grown to test their suitability for further production. The documented situation captures a changepoint in the agricultural system and the beginning of the systematic introduction of various exotic crops and spices at end of the 17th century. During the 18th century, the transformation of agriculture and forestry culminated, partly influenced by the spread of the Enlightenment ideas and new technologies.

## 5 Conclusion

Research of the production centre at Švamberk provided a detailed insight into the economic structure of the basic unit of the modern central European manorial system: the manor farm.

Dendrochronological analysis of wooden timbers revealed the history of building activity (spanning from the mid-17th century to the mid-19th century), as well as the changes in the species used for construction wood over time. Forest management practices are evident in the ongoing changes in timber production, which was reflected in the dendrochronologically dated timbers. Strontium analysis, combined with tree ring sequences showed diverse wood provenance and variations in the species origin. The shift from the predominant use of fir wood to spruce wood might be partially caused by the changes in modern forestry management and natural succession in the vicinity of Švamberk manor farm; however, it was probably related to advancements in river transport of the spruce timbers from the higher altitudes. This is supported by the different values of ^87^Sr/^86^Sr and variability within the dendrochronological sequences. These findings, however, require further testing with additional analysed samples in future studies.

Tree ring analysis played a crucial role in dating the intact sediments of the vault infills, which contained an exceptionally large number of plant remains which are more than representative of the agricultural practices at the manor farm (81,892 individual specimens). Many species of economically significant plants, such as tobacco, maize and sorghum, had been sparsely, if at all recorded in archaeological material from central Europe prior to this discovery.

Part of the archaeobotanical collection included cereal processing waste primarily intended for brewery and food production. Animal husbandry was also a significant part of the manor farm's economy, utilising cereals for that purpose.

Survey of the farmyard thus capture the changepoint of European agriculture and landscape influenced by the globalisation, enlightenment philosophy and capitalism when the traditional crops started to be complemented by the crops from Americas, Africa and Asia. First only in small (experimental) scale in the gardens.

The Manor farm served as a temporary residence for the nobility, leading to the production of the supplies for the kitchen of the prince grown on site. The introduction of plant species from the Americas and Africa caused a major shift in agriculture. Compared to the Mediterranean area in central Europe, neophyte taxa were introduced into agriculture relatively late, in most cases probably in the 17th century as evidenced by assemblages from the Švamberk manor farm. Many of these plants belonged to ornamental species. Additionally, a portion of plants used in agricultural production was foraged in the forest.

## CRediT authorship contribution statement

**Libor Vobejda:** Writing – review & editing, Writing – original draft, Visualization, Methodology, Investigation, Formal analysis. **Tereza Šálková:** Writing – review & editing, Writing – original draft, Validation, Methodology, Investigation, Formal analysis, Data curation, Conceptualization. **Yulia V. Erban Kochergina:** Writing – review & editing, Methodology, Investigation, Formal analysis, Data curation. **Jan Altman:** Writing – review & editing, Validation. **Zuzana Thomová:** Resources, Project administration.

## Data availability statement

All data used in the study are included within the article and supplementary materials. Raw archaeobotanical data and dating of archaeological features is available in (.xlsx) sheets. Detrended dendrochronological measurements are available in Tucson files (.rwl).

## Funding

Libor Vobejda was supported by the Grant Agency 10.13039/100010100USB (CZE) No. 095/2022/H (V. Bůžek) Increasing the theoretical and methodological competences of students of doctoral study programmes in historical sciences.

Jan Altman was supported by the long-term research development project No. RVO 67985939 of the Czech Academy of Sciences.

## Declaration of competing interest

The authors declare that they have no known competing financial interests or personal relationships that could have appeared to influence the work reported in this paper.

## Footnotes

1 The period of the late 17th and 18th centuries was characterized by a stream of thought associated with humanism, scientific advancements, and the development of aesthetics, particularly concerning the perception of nature [17].

2 In the 17th and 18th centuries, an economic ideology known as mercantilism was prevalent among political elites. This doctrine emerged in a polarized Europe, where economic theorists viewed international trade as a form of competition among great powers. The fundamental goal was to achieve economic self-sufficiency for the state and maximize exports [26].

3 The wooden pipeline was dated to 1752 (see Ciglbauer et al. [55]).

4 The waney edge is title used for description of the tree last increment ring located under the bark. If waney edge is present, the felling of the tree is possible to date with subannual accuracy (see Refs. [91,143]).

5 As prominent diplomats and high-ranking officials [62], the owners of the estates were undoubtedly familiar with the prevailing economic doctrine of mercantilism, which emphasised the minimisation of imports in favour of exports [24,26,27].

6 Request for a deputation submitted by the free journeyman Zelenka in 1720, who worked on the roofs of the Švamberk farm. Deposited: SOA Třeboň, department Český Krumlov, sign. B 7D 8, s.f.

## References

[1] Lamentowicz M., Marcisz K., Guzowski P., Gałka M., Diaconu A.-C., Kołaczek P. (2020). How Joannites’ economy eradicated primeval forest and created anthroecosystems in medieval Central Europe. Sci. Rep..

[2] Wensman S.M., Shiel A.E., McConnell J.R. (2022). Lead isotopic fingerprinting of 250-years of industrial era pollution in Greenland ice. Anthropocene.

[3] Danneyrolles V., Dupuis S., Fortin G., Leroyer M., de Römer A., Terrail R., Vellend M., Boucher Y., Laflamme J., Bergeron Y., Arseneault D. (2019). Stronger influence of anthropogenic disturbance than climate change on century-scale compositional changes in northern forests. Nat. Commun..

[4] Pokorná A., Kočár P., Novák J., Šálková T., Žáčková P., Komárková V., Vaněček Z., Sádlo J. (2018). Ancient and early medievalman-made habitats in the Czech republic. Preslia.

[5] Muir R. (1999). Approaches to Landscape.

[6] Kučera Z. (2009). Landscape in Czech geography and the problem of relevance of Anglo-American human geography approaches. Geografie.

[7] Dolejš M., Nádvorník J., Raška P., Riezner J. (2019). Frozen histories or narratives of change? Contextualizing land-use dynamics for conservation of historical rural landscapes. Environ. Manag..

[8] Izakovičová Z., Petrovič F., Pauditšová E. (2021). The impacts of urbanisation on landscape and environment: the case of Slovakia. Sustainability.

[9] Santoro A. (2024). Why traditional rural landscapes are still important to our future. Landsc. Ecol..

[10] Kyryanová P. (2022).

[11] Iordachi C., Bauerkämper A. (2014). The Collectivization of Agriculture in Communist Eastern Europe: Comparison and Entanglements.

[12] Mohr F., Diogo V., Helfenstein J., Debonne N., Dimopoulos T., Dramstad W., García-Martín M., Hernik J., Herzog F., Kizos T., Lausch A., Lehmann L., Levers C., Pazur R., Ruiz-Aragón V., Swart R., Thenail C., Ulfeng H., Verburg P.H., Williams T., Zarina A., Bürgi M. (2023). Why has farming in Europe changed? A farmers’ perspective on the development since the 1960s. Reg. Environ. Change.

[13] Andresková E. (1999). Vývoj lesů a lesního hospodářství na Hlubocku. Archeologické památky okresu České Budějovice.

[14] Kyryanová P. (2020).

[15] Ivanega J. (2014). Hluboká – lovecký zámek Ohrada a schwarzenberská sídla na panství Hluboká nad Vltavou.

[16] Stibral K. (2007). Je les krásný? Estetické vnímání lesa a přírody v průběhustaletí, Dějiny a Současnost. Kulturně Historická Revue.

[17] Symes M. (2016). ENLIGHTENMENT, the “natural” garden and BROWN. Garden History.

[18] Preusz M., Kodýdková K., Kočár P., Vaněček Z. (2015). Exotic spices in flux: archaeobotanical material from medieval and early modern sites of the Czech lands (Czech republic). Interdisciplinaria Archaeologica - Natural Sciences in Archaeology, VI.

[19] Čulíková V. (2013). Moderní sortiment užitkových rostlin v barokové jímce v Thunovské ulici čp. 192 na Malé Straně v Praze. Staletá Praha.

[20] Tenaillon M.I., Charcosset A. (2011). A European perspective on maize history. C R Biol.

[21] Rapp R.T. (1975). The unmaking of the mediterranean trade hegemony: international trade rivalry and the commercial revolution. J. Econ. Hist..

[22] Hyden-Hanscho V., Hats Beaver, Hyden-Hanscho V., Pieper R., Stangl W., Judith Ann Carney (2013). Cultural Exchange and Consumption Patterns in the Age of Enlightenment: Europe and the Atlantic World.

[23] Topolski J. (1974). The Manorial Economy in Early-Modern East-Central Europe.

[24] Turzyński M. (2011). Bookkeeping in Manor Farms of Polish Gentry in 17th Century. Eurasian J. Bus. Econ..

[25] Slavíčková P. (2022). Early bookkeeping handbooks from central Europe: a case study of the Czech lands. Account. Hist. J..

[26] Cole C.W. (1939).

[27] von Höornigk P. (1964).

[28] Kosňovská J. (2011). Archeobotanická analýza výplně klenebního výsypu z vladislavského sálu na pražském hradu.

[29] Beneš J., Čulíková V., Kosňovská J., Frolík J., Matiášek J. (2012). New plants at Prague Castle and hradčany in the early Modern Period: a history of selected species. Interdisciplinaria Archeol. Nat. Sci. Archaeol..

[30] Hrdlička J. (2000). Hodovní stůl a dvorská společnost: strava na raně novověkých aristokratických dvorech v českých zemích 1550-1650, České Budějovice.

[31] Lugli F., Brunelli D., Cipriani A., Bosi G., Traversari M., Gruppioni G. (2017). C4-Plant foraging in northern Italy: stable isotopes, Sr/Ca and Ba/Ca data of human osteological samples from roccapelago (16th–18th centuries AD). Archaeometry.

[32] Kamen H. (1968). The economic and social consequences of the thirty years, war. Past Present.

[33] Čechura J. (2020).

[34] Knoz T. (2006). Pobělohorské konfiskace. Moravský průběh, středoevropské souvislosti, obecné aspekty.

[35] Válka J. (1983). Česká Společnost V 15. - 18. Století. II. Bělohorská Doba. Společnost a Kultura Manýrismu.

[36] Asch R.G. (1988). Estates and princes after 1648: the consequences of the thirty years war. Ger. Hist..

[37] Čechura J. (2016). Hospodářský pragmatik Jan Adolf ze Schwarzenberka a panství Hluboká nad Vltavou v letech 1661–1681. Jihočeský Sborník Historický.

[38] Valenta A. (2011). Lesk a Bída Barokní Aristokracie.

[39] Vařeka M. (2018). Režijní Velkostatek Na Předbělohorské Moravě.

[40] North D.C., Paul Thomas R. (1971). The rise and fall of the manorial system: a theoretical model. J. Econ. Hist..

[41] Dribe M., Olsson M., Svensson P., Angell J. (2012). Manorialism and risk management in pre-industrial society: Sweden in the eighteenth and nineteenth centuries, Annales. Histoire. Sciences Sociales.

[42] Stolička O. (2015). Zásobování Schwarzenberské Domácnosti Ve Druhé Polovině 17.

[43] Petráň J. (1963). Zemedelská výroba v Cechách v druhé polovine 16.

[44] Andreska J. (1997).

[45] Čapek L. (2011). Dvě zaniklé středověké vesnice ve Velechvínském polesí, okr. České Budějovice. Archeologické Výzkumy v Jižních Čechách.

[46] Čejková A., Kyncl T., Kolář T. (2005). Využití jedlového dřeva v dřevěných konstrukcích historických staveb. Sborník Referátů Konference Jedle Bělokorá 2005 (European Silver Fir - 2005).

[47] Stehlíková E. (2014).

[48] Hladík H. (2021). Schwarzenberský plavební kanál v zrcadle historických dokumentů. Vojenské lesy a statky ČR.

[49] Záloha J. (1973). Josef Rosenauer - Šumavský plavební kanál.

[50] Kolář T., Dobrovolný P., Szabó P., Mikita T., Kyncl T., Kyncl J., Sochová I., Rybníček M. (2021). Wood species utilization for timber constructions in the Czech lands over the period 1400–1900. Dendrochronologia.

[51] Rasmussen C.P. (2010). Innovative Feudalism. The development of dairy farming and “Koppelwirtschaft” on manors in Schleswig-Holstein in the seventeenth and eighteenth centuries,The. Agric. Hist. Rev..

[52] Ryantová M. (2014). Vrchnostenské hospodaření na panství Vysoký Chlumec v 17. a na počátku 18. století, Pelhřimov.

[53] Statuto D., Cillis G., Picuno P. (2017). Using historical maps within a GIS to analyze two centuries of rural landscape changes in southern Italy. Land.

[54] Suchan J. (1978). Zpravodaj Ševětínska.

[55] Ciglbauer J., Vobejda L., Figura J., Hieke E., Krajíc R., Šálková T. (2023). Využívání vody na šlechtických hospodářských dvorech na příkladu dendroarcheologie dvora Švamberku, okres České Budějovice. Archeologické Výzkumy v Jižních Čechách.

[56] Chocholáč B. (1994). Collatovské kuchyňské účty z 2. poloviny 17. století : příspěvek ke studiu šlechtické každodennosti. Sb. Pr. Filoz. Fak. Brnenské Univ..

[57] Jacomet S., Kreutz A. (1999). Archäobotanik – Aufgaben, Methoden und Ergebnisse vegetations- und agrargeschichtlicher Forschung.

[58] Reynolds A.C., Betancourt J.L., Quade J., Jonathan Patchett P., Dean J.S., Stein J. (2005). 87Sr/86Sr sourcing of ponderosa pine used in Anasazi great house construction at Chaco Canyon, New Mexico. J. Archaeol. Sci..

[59] D’Andrea R., Corona C., Poszwa A., Belingard C., Domínguez-Delmás M., Stoffel M., Crivellaro A., Crouzevialle R., Cerbelaud F., Costa G., Paradis-Grenouillet S. (2023). Combining conventional tree-ring measurements with wood anatomy and strontium isotope analyses enables dendroprovenancing at the local scale. Sci. Total Environ..

[60] Čechura J. (2021).

[61] Urban J. (1999). Dvůr Švamberk u Ševětína. Zprávy Památkové Péče.

[62] Smíšek R. (2009). Císařský dvůr a dvorská kariéra Ditrichštejnů a Schwarzenberků za vlády Leopolda I. České Budějovice.

[63] Rameš V., Bezecný Z., Gaži M., Putna M.C. (2008). Schwarzenbergové V České a Středoevropské Kulturní Historii.

[64] Křivánek J. (2012). Rybníky V České Republice.

[65] Ciglbauer J., Hieke E., Šálková T., Vobejda L. (2021). Milíře a dřevěné uhlí ve Velechvínském polesí a okolí Chotýčan. Archeologické Výzkumy v Jižních Čechách.

[66] Jírů R. (2012). Počátky dolování hnědého uhlí na Postoloprtsku, 1719-1766., Z Dějin Hornictví. Těžba Surovin v Proměnách Času.

[67] Lange F. (2004).

[68] Novotná M. (2017).

[69] Trapp R. (1995). Schriften Des Vereines Zur Verbreitung Naturwissenschaftlicher Kenntnisse in Wien.

[70] Čufar K., Bizjak M., Kitek Kuzman M., Merela M., Grabner M., Brus R. (2014). Castle Pišece, Slovenia – building history and wood economy revealed by dendrochronology, dendroprovenancing and historical sources. Dendrochronologia.

[71] Laurincová S. (2009).

[72] Jacomet S., Greig J. (2006). Identification of cereal remains from archaeological sites.

[73] Beneš J., Kolář T., Čejková A., Hašek V., Nekuda R., Ruttkay M. (2006). Ve Službách Archeologie. , Muzejní a Vlastivědná Společnost V Brně.

[74] Beneš J., Kaštovský J., Kočárová R., Kočár P., Kubečková K., Pokorný P., Starec P. (2002). Archaeobotany of the Old Prague Town defence system, Czech Republic: archaeology, macro-remains, pollen, and diatoms. Veg. Hist. Archaeobotany.

[75] de Vareilles A., Pelling R., Woodbridge J., Fyfe R. (2021). Archaeology and agriculture: plants, people, and past land-use. Trends Ecol. Evol..

[76] Holzer S.M., Köck B. (2009). Investigations into the structural behavior of German baroque timber roofs. Int. J. Architect. Herit..

[77] Vinař J. (2010). Historické krovy: Typologie, průzkum, opravy.

[78] Schweingruber F.H. (1978). Mikroskopische holzanatomie.

[79] Maxwell R.S., Larsson L.-A. (2021). Measuring tree-ring widths using the CooRecorder software application. Dendrochronologia.

[80] Baillie M.G.L., Pilcher J.R. (1973). A simple crossdating program for tree-ring research. Tree-Ring Bull..

[81] Becker B., Giertz-Siebenlist V. (1970). Eine über 1100jährige mitteleuropäische Tannenchronologie1)1)Unserem verehrten Lehrer, Herrn Professor Dr. Dr. h. c. Dr. h. c. Bruno Huber zur Vollendung des 70. Lebensjahres gewidmet. Flora.

[82] Kyncl T. (2007). Dub-ČR07. Master Chronol..

[83] Kyncl T. (2005). Jedle ČR 2005. Master Chronol..

[84] Kyncl T. (2003). Smrk-Čechy 2003. Master Chronol..

[85] Kyncl T. (2003). Borovice-Čechy 2003. Master Chronol..

[86] Kolář T. (2005). NTpcab05-sta, Master Chronology.

[87] Čejková A., Kolář T. (2009). Extreme radial growth reaction of Norway spruce along an altitudinal gradient in the Šumava mountains. GEOCHR.

[88] Becker B., Giertz-Siebenlist V. (1970). Eine über 1100-jährige mitteleuropische Tannenchronologie. Flora.

[89] Čermák P., Rybníček M., Žid T., Steffenrem A., Kolář T. (2019). Site and age-dependent responses of Picea abies growth to climate variability. Eur. J. For. Res..

[90] Baillie M.G.L. (1982). Tree-ring Dating and Archaeology.

[91] Fritts H.C. (1976). http://www.sciencedirect.com:5070/book/9780122684500/tree-rings-and-climate.

[92] Haneca K., Wazny T., Van Acker J., Beeckman H. (2005). Provenancing Baltic timber from art historical objects: success and limitations. J. Archaeol. Sci..

[93] Jansma E., Haneca K., Kosian M. (2014). A dendrochronological reassessment of three Roman boats from Utrecht (The Netherlands): evidence of inland navigation between the lower-Scheldt region in Gallia Belgica and the limes of Germania inferior. J. Archaeol. Sci..

[94] Erban Kochergina Y.V., Novak M., Erban V., Stepanova M. (2021). 87Sr/86Sr isotope ratios in trees as an archaeological tracer: limitations of linking plant-biomass and bedrock Sr isotope signatures. J. Archaeol. Sci..

[95] Novák M., Andronikov A.V., Holmden C., Erban Kochergina Y.V., Veselovský F., Pačes T., Vítková M., Kachlík V., Šebek O., Hruška J., Štěpánová M., Čuřík J., Přechová E., Fottová D., Andronikova I.E., Erban V., Koubová M., Vostrá I., Housková M., Komárek A. (2023). δ26Mg, δ44Ca and 87Sr/86Sr isotope differences among bedrock minerals constrain runoff generation in headwater catchments: an acidified granitic site in Central Europe as an example. Catena.

[96] Madgwick R., Lewis J., Grimes V., Guest P. (2019). On the hoof: exploring the supply of animals to the Roman legionary fortress at Caerleon using strontium (87Sr/86Sr) isotope analysis. Archaeol. Anthropol. Sci..

[97] Erban Kochergina Y.V., Erban V., Hora J.M. (2022). Sample preparation and chromatographic separation for Sr, Nd, and Pb isotope analysis in geological, environmental, and archaeological samples. J. Geosci..

[98] Hajnalová E. (1999). Archeobotanika pestovaných rastlín.

[99] Berggren G. (1981).

[100] Anderberg A.-L. (1994).

[101] Fenner M., Cappers R.T.J., Bekker R.M., Jans J.E.A. (2006). http://www.seedatlas.nl.

[102] Ter Braak C.J.F., Šmilauer P. (2012). CANOCO reference manual and CanoDraw for Windows user’s guide: Software for Canonical Community Ordination (version 4.5).

[103] Hajdas I., Ascough P., Garnett M.H., Fallon S.J., Pearson C.L., Quarta G., Spalding K.L., Yamaguchi H., Yoneda M. (2021). Radiocarbon dating. Nat. Rev. Methods Primers.

[104] Libby W.F., Anderson E.C., Arnold J.R. (1949). Age determination by radiocarbon content: world-wide assay of natural radiocarbon. Science.

[105] Fouédjeu L., Saulnier M., Lejay M., Dušátko M., Labbas V., Jump A.S., Burri S., Buscaino S., Py-Saragaglia V. (2021). High resolution reconstruction of modern charcoal production kilns: an integrated approach combining dendrochronology, micromorphology and anthracology in the French Pyrenees. Quat. Int..

[106] Novak M., Holmden C., Farkas J., Kram P., Hruska J., Curik J., Veselovsky F., Stepanova M., Erban Kochergina Y.V., Erban V., Andronikov A., Sebek O., Koubova M., Bohdalkova L., Vitkova H. (2020). Magnesium and calcium isotope systematics in a headwater catchment underlain by amphibolite: constraints on Mg–Ca biogeochemistry in an atmospherically polluted but well-buffered spruce ecosystem (Czech Republic, Central Europe). Catena.

[107] Novak M., Holmden C., Farkaš J., Kram P., Hruska J., Curik J., Veselovsky F., Stepanova M., Erban Kochergina Y.V., Erban V., Fottova D., Simecek M., Bohdalkova L., Prechova E., Voldrichova P., Cernohous V. (2020). Calcium and strontium isotope dynamics in three polluted forest ecosystems of the Czech Republic, Central Europe. Chem. Geol..

[108] Janoušek V., Vrána S., Erban V. (2002). Petrology, geochemical character and petrogenesis of a variscan post-orogenic granite: case study from the Ševětín Massif, moldanubian batholith, southern bohemia. J. Czech Geol. Soc..

[109] Macků P. (2014). Kosňovská Jitka, Archeologický a archeobotanický výzkum raně novověké studny z Telče – Štěpnic. Archeologické Výzkumy Na Vysočině.

[110] MattioIi P.O. (1982). Herbář, Jinak Bylinář.

[111] Stehlíková E. (2013). Dendrochronologická Analýza Historického Materiálu Z Vybraných Staveb Podél Horního Toku Vltavy, Unpublished MA Thesis.

[112] Blažková T., Blažková T., Červinková P. (2015). Krajina Jako Antropologická Čítanka.

[113] Leuschner C., Meier I.C. (2018). The ecology of Central European tree species: trait spectra, functional trade-offs, and ecological classification of adult trees. Perspect. Plant Ecol. Evol. Systemat..

[114] Eckstein D., Krause C. (1989). Dendroecologlcal studies on spruce trefs to monitor environmental changes around hamburg. IAWA J..

[115] Šálková T., Vobejda L., Chvojka O., Beneš J., Vondrovský V., Kuna M., Křivánek R., Menšík P., Novák J. (2022). Extensive archaeobotanical data estimate carrying capacity, duration, and land use of the Late Bronze Age settlement site Březnice (Czech Republic). Sci. Rep..

[116] Kolář T. (2006). Čejková Alžběta, Kyncl Tomáš, Xylotomic and dendrochronological analyses in archaeology: changes in the composition type of wood in Prague and in Southern Bohemia. Ve Službách Archeologie.

[117] Holec F. (1971). Obchod s dřívím v Praze ve 14. – 17. století. Pražský Sborník Historický.

[118] Kaplan Z., Danihelka J., Šumberová K., Prančl J., Velebil J., Dřevojan P., Ducháček M., Businský R., Řepka R., Maděra P., Galušková H., Wild J., Brůna J. (2023). Distributions of vascular plants in the Czech Republic. Part 12. Preslia.

[119] Strnadová D. (2011). Kukuřice – dar z Nového světa. Národní zemědělské muzeum,.

[120] Stitzer M.C., Ross J. (2018). Ibarra, Maize domestication and gene interaction. New Phytol..

[121] Gore M.A., Chia J.M., Elshire R.J., Sun Q., Ersoz E.S., Hurwitz B.L., Peiffer J.A., McMullen M.D., Grills G.S., Ross-Ibarra J., Ware D.H., Buckler E.S. (2009). A first-generation haplotype map of maize. Science.

[122] Andrews J. (1993). Diffusion of mesoamerican food complex to southeastern Europe. Geogr. Rev..

[123] Petrović G.G. (2019). Maize cultivation in Serbia: a historical perspective. Istor. Cas..

[124] Harlan J.R., Wet J.M.J. (1971). Toward a rational classification of cultivated plants. Taxon.

[125] Vychronová M. (2012). Západočeská Univerzita V Plzni.

[126] Měrková J. (2013).

[127] Řeháková Z., Karlíčková J., Jahodář L. (2008). SLUNEČNICE ROČNÍ (Helianthus annuus L.)-Obsahové látky A biologická aktivita. Chemické listy.

[128] Čeřovský J. (1952). Zajímavý případ zplanění zeravu východního Thuja orientalis L. Čs. Bot. Listy,.

[129] Kubát K. (2002).

[130] Čulíková V. (1995). Zpráva o prvním archeobotanickém nálezu tabáku (r. Nicotiana L.) ve střední Evropě. Archaeologia Historica.

[131] Pejml K. (1947).

[132] Vyšohlíd M. (2009). Kramické dýmky v archeologických nálezech a jejich vypovídací možnosti. Archeologie ve Středních Čechách.

[133] Mehler N. (2018). Clay tobacco-pipe research and historical archaeology in Germany, a difficult relationship. Hist. Archaeol..

[134] Bezděk A., Houfková P., Kovačiková L., Šáková T., Chvojka O., John J., Thomová Z. (2016). Bošilecký Poklad.

[135] Olah B., Boltižiar M., Gallay I. (2009). Transformation of the Slovak cultural landscape since the 18th cent. And its recent trends. J. Landsc. Ecol..

[136] Antrop M. (2005). Why landscapes of the past are important for the future. Landsc. Urban Plann..

[137] Kędziora A., Zerihun Negussie Y., Tenaw Asres M., Zalewski M. (2011). Shaping of an agricultural landscape to increase water and nutrient retention. Ecohydrol. Hydrobiol..

[138] Šťastná M., Vaishar A. (2020). Values of rural landscape: the case study Chlum u Třeboně (Bohemia). Land Use Pol..

[139] Pokorná A., Houfková P., Novák J., Bešta T., Kovačiková L., Nováková K., Zavřel J., Starec P. (2014). The oldest Czech fishpond discovered? An interdisciplinary approach to reconstruction of local vegetation in mediaeval Prague suburbs. Hydrobiologia.

[140] Jan Ivanega, Wohl (2016).

[141] Šálková T., Beneš J., Komárková V., Vaněček Z. (2012). History of barley (Hordeum vulgare) in Central Europe according to archaeobotanical findings. Kvasny Prumysl.

[142] Šálková T., Chvojka O., Hlásek D., Jiřík J., John J., Novák J., Kovačiková L., Beneš J. (2019). Crops along the trade routes? Archaeobotany of the Bronze Age in the region of South Bohemia (Czech Republic) in context with longer distance trade and exchange networks. Archaeol Anthropol Sci..

[143] Fowler A. (1988). Climatic reconstruction from tree rings. Weather Clim..

